# Sympathetic nerve block as an add-on therapy for intervention and prevention of cerebral vasospasm after subarachnoid hemorrhage

**DOI:** 10.3389/fneur.2025.1571550

**Published:** 2025-06-06

**Authors:** Zhaoquan Wang, Jianqiang Li

**Affiliations:** Department of Critical Care Medicine, Weifang People's Hospital, Weifang, Shandong, China

**Keywords:** cerebral vasospasm, subarachnoid hemorrhage, sympathetic nervous system, stellate ganglion block, ROS

## Abstract

Cerebral vasospasm is a major complication after subarachnoid hemorrhage (SAH) and is an important factor leading to disability and mortality in patients. Cerebral vasospasm involves cerebral artery stenosis and leads to delayed cerebral ischemia, further exacerbating brain damage. The pathophysiology of cerebral vasospasm is multifactorial, involving a complex interaction between fragmented red blood cell metabolism, endothelial dysfunction, and hyperresponsive contraction of smooth muscle cells. Recent studies have highlighted the important role of the sympathetic nervous system (SNS) in mediating and exacerbating cerebral vasospasm. Sympathetic activation affects vascular tone and contributes to the development of vasospasm after SAH. Stellate ganglion block (SGB) has been reported to have a protective effect in patients at risk for vasospasm after SAH due to reduced sympathetic activity. This review aims to explore the current understanding of the relationship between sympathetic activity and cerebral vasospasm, investigate the molecular mechanisms involved, clinical implications, and potential therapeutic strategies targeting sympathetic modulation.

## 1 Introduction

Subarachnoid hemorrhage (SAH) is a life-threatening neurological condition, most commonly caused by the rupture of an intracranial aneurysm, leading to the extravasation of blood into the subarachnoid space. Accumulating evidence indicates that early brain injury (EBI) and delayed cerebral ischemia (DCI) are two major pathophysiological processes, playing pivotal roles in the progression of SAH (1). Following aneurysmal rupture, the rapid influx of blood into the subarachnoid space results in a sharp increase in intracranial pressure, which consequently reduces cerebral perfusion pressure (2). This reduction leads to cerebral ischemia and hypoxia, thereby initiating EBI. EBI typically occurs within the first 72 h after SAH onset and is characterized by neuronal cell death, blood–brain barrier disruption, cerebral edema, acute cerebral vasospasm, and microvascular dysfunction (3). Elevated levels of endothelin-1 and oxygenated hemoglobin released into the subarachnoid space further activate apoptotic and inflammatory pathways, exacerbating vasospasm, microthrombosis, and disturbances in cerebral blood flow (4). These changes ultimately contribute to cerebral infarction and neurological impairment. EBI not only causes direct injury to neural tissue but also predisposes patients to secondary complications such as DCI (1). As the condition progresses, impairments in cerebral autoregulation, microcirculatory dysfunction, and sustained blood–brain barrier damage contribute to the development of DCI. Clinical studies have demonstrated that ~70% of SAH patients experience cerebral vasospasm by the third day, with a peak incidence between days 7 and 8 (5). Moreover, about 30% of patients with aneurysmal SAH develop DCI between days 4 and 10. Cerebral vasospasm is considered a major contributor to DCI, leading to cerebral infarction and persistent neurological deficits, and is regarded as a critical determinant of poor clinical outcomes in SAH patients (6).

Cerebral vasospasm is a major cause of delayed ischemic injury, typically occurring 3–14 days following SAH, and continues to be a significant contributor to both morbidity and mortality in SAH patients (6, 7). Cerebral vasospasm is characterized by a prolonged narrowing of the cerebral arteries, resulting in a reduction of cerebral blood flow, which can lead to ischemic damage and subsequent neurological deficits (8). Despite extensive research into the roles of erythrocyte degradation products (9–11), endothelial dysfunction (12, 13), and smooth muscle hypercontraction (14, 15) as key mediators of vasospasm, the precise mechanisms underlying cerebral vasospasm remain incompletely understood. To date, treatments for vasospasm include nimodipine (16), the combination of induced hypertension, hypervolemia, and hemodilution (triple-H therapy) (17), and interventional neuroradiological procedures such as transluminal angioplasty (18) or intra-arterial vasodilators (19), which are associated with severe side effects such as hypotension. Magnesium, statins, endothelin antagonists, and fibrinolytic therapy (20, 21) are under investigation, but large-scale trials are needed to demonstrate their effectiveness. Therefore, there is currently no effective method for the prevention and treatment of cerebral vasospasm.

The sympathetic nervous system (SNS), a key component of the autonomic nervous system, plays a crucial role in regulating vascular tone (22). Its activation in response to stress or injury contributes to the onset and exacerbation of vasospasm. Increasing evidence suggests that the sympathetic nervous system plays a critical role in the pathophysiology of cerebral vasospasm (23, 24). The interaction between the SNS and cerebral vasospasm is a complex and multifactorial process. Following SAH, the stress response and inflammatory cascade activate the SNS, leading to the release of norepinephrine from both sympathetic nerve terminals and the adrenal medulla (25). Several studies have shown that sympathetic nerve is activated after subarachnoid bleeding (25–27). Therefore, selective blockade of sympathetic nerves has become a new and promising method for the treatment and prevention of cerebral vasospasm. Stellate ganglion block (SGB) has been proposed as a simple, minimally invasive technique that can effectively improve cerebral perfusion and prevent cerebral vasospasm by relieving symptomatic cerebral vasospasm (26–29). By inhibiting sympathetic nerve activity, SGB potentially relieves vasoconstriction and improves blood flow in cerebral vasospasm (30, 31).

This review aims to examine the role of the SNS in the development of cerebral vasospasm following SAH. We will explore how sympathetic activation contributes to the pathophysiology of cerebral vasospasm, the underlying mechanisms mediating these effects, and the potential therapeutic implications of modulating sympathetic activity as a strategy for preventing or treating cerebral vasospasm in SAH patients. By investigating the interactions between the SNS and the cerebral vasculature, this review seeks to provide insights into novel approaches that could improve outcomes for SAH patients, who are at increased risk of the severe consequences associated with cerebral vasospasm. Meanwhile, we discuss SGB as an add-on therapy for intervention and prevention of cerebral vasospasm after SAH.

## 2 Pathophysiology of cerebral vasospasm

**Figure 1:**
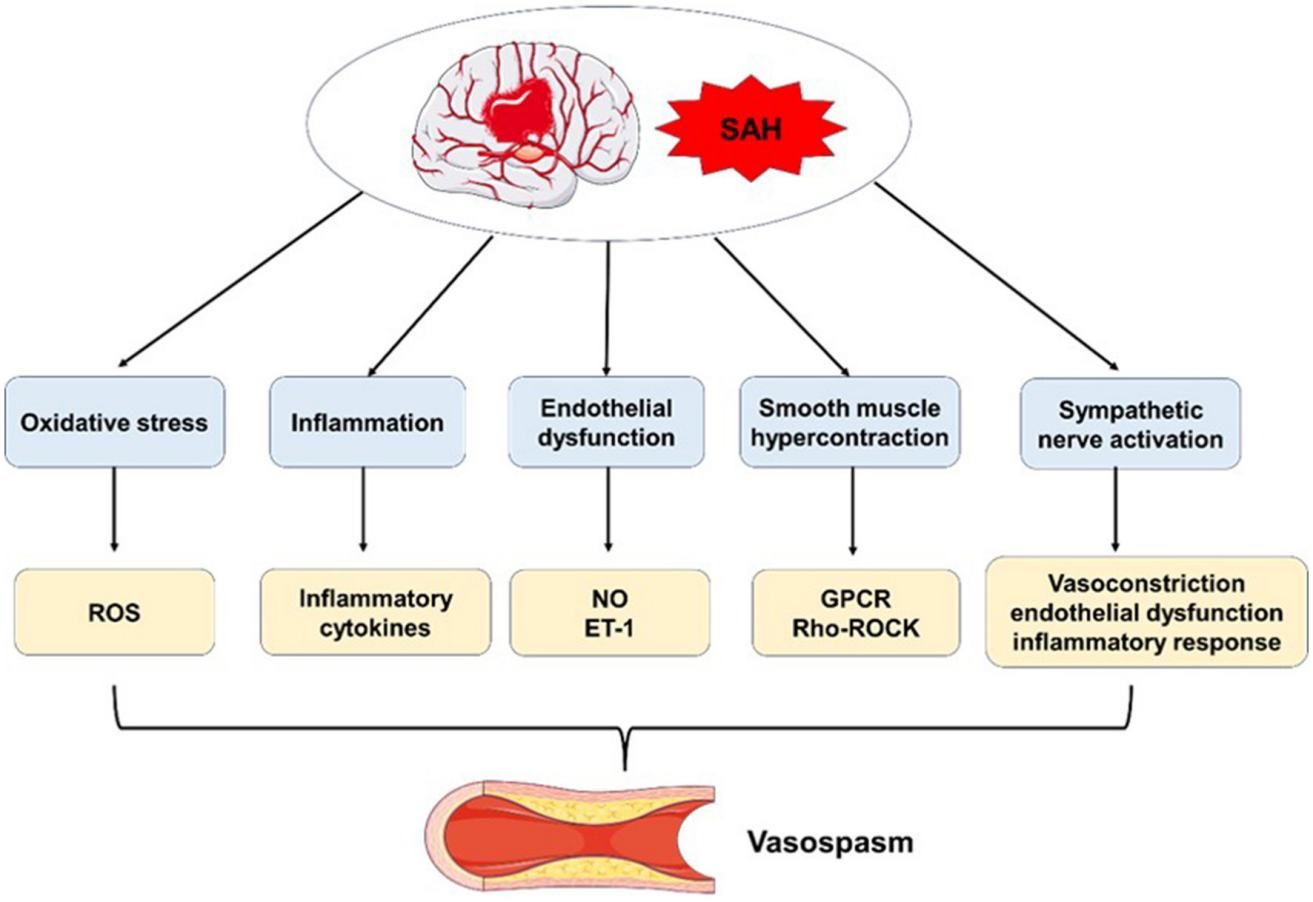
The pathophysiology of cerebral vasospasm after SAH.

Cerebral vasospasm is a significant contributor to death and disability in patients with SAH (32). Despite ongoing advancements in medical technology, no effective treatment strategy for cerebral vasospasm has been established to date. Many studies have demonstrated that delayed ischemia resulting from cerebral vasospasm is a major cause of morbidity and mortality following SAH (33–36). Statistical analyses of patient data indicate that the overall incidence of angiographic vasospasm after SAH is 43.3%, with the incidence rising to 67.3% when angiography is performed at the time of maximum spasm (37). Moreover, 32.5% of patients exhibit symptomatic vasospasm or delayed ischemic deficits (38). While the precise pathophysiology of cerebral vasospasm remains under investigation, several key factors are believed to contribute to its development, including oxidative stress, inflammation, endothelial dysfunction, smooth muscle hypercontraction, and sympathetic nervous system activation (Figure 1).

### 2.1 Oxidative stress and cerebral vasospasm

Oxidative stress arises from an imbalance between the generation of reactive oxygen species (ROS) and antioxidant defense mechanisms. ROS affects cerebrovascular smooth muscle tone, permeability, and brain autoregulation (39) through various biochemical pathways, with this effect potentially being more pronounced in the presence of cerebrovascular disease. After SAH, excessive ROS production originates from multiple sources, including disruption of mitochondrial respiration, upregulation of enzyme pathways, degradation of extracellular hemoglobin, and inhibition of intrinsic antioxidant systems (40). Studies have demonstrated that ROS induce vasoconstriction by strongly inhibiting endothelial nitric oxide (NO)-mediated vasodilation (41, 42). Results from numerous *in vitro* and *in vivo* animal studies indicate that O^2−^ has a biphasic effect on cerebrovascular vessels, depending on its concentration (43). At low concentration, O^2−^ induces vasodilation, while at high concentration, it causes vasoconstriction. Additionally, O^2−^ can react with arachidonic acid and other unsaturated fatty acids, leading to the formation of isoprostanes, which are potent vasoconstrictors that may reduce cerebral blood flow (39). Moreover, the production rates of ROS and antioxidants are influenced by oxygenation levels, each following distinct kinetics. Under pathological conditions, such as acute brain injury or SAH, ROS levels increase significantly and persist over prolonged periods (44). There are multiple sources of excess free radical production after SAH, including disruption of mitochondrial respiration and extracellular hemoglobin. Moreover, free radical-producing enzymes such as inducible nitric oxide synthase (iNOS), xanthine oxidase, NADPH oxidase (NOX), and enzymes involved in arachidonic acid metabolism are upregulated. In addition, intrinsic antioxidant systems such as superoxide dismutase (SOD) and glutathione peroxidase (GSH-Px) are inhibited (44).

Studies have shown that oxidative stress is one of the factors that cause vasospasm after hemorrhage (45–47). Maeda et al. (48) found that exposure of bovine middle cerebral artery strips to oxidative stress inhibited bradykinin-induced endothelium-dependent relaxation. Oxidative stress stimulates smooth muscle cell proliferation and hypertrophy (49) and induces endothelial cell apoptosis. In addition, increased levels of superoxide anions in cerebrospinal fluid after SAH are associated with cerebral vasospasm (50). Research is continuing to further elucidate how oxidative stress alters cerebral vasoconstrictor responses.

### 2.2 Inflammation and cerebral vasospasm

Much evidence suggests that inflammatory response plays a key role in the development and maintenance of cerebral vasospasm after SAH (51–53). Data from Bowman et al. (54) showed that inflammatory cytokines, particularly IL-6, are associated with the development of vasospasm in a rat femoral artery model. Lu et al. (55) found that mRNA and protein levels of monocyte chemoattractant protein-1 (MCP-1), a potent macrophage chemoattractant, increased in parallel with the development of cerebral vasospasm in a rat double hemorrhage model, suggesting that the use of specific MCP-1 antagonists may be useful in preventing SAH-induced vasospasm. Simvastatin administration after SAH can reduce vasospasm, and perivascular granulocyte migration was found to be reduced after 72 h of SAH, suggesting that simvastatin may relieve vasospasm through its anti-inflammatory effect (56). Another study from Zhou et al. (57) demonstrated that the mRNA levels of TNF-a, IL-1b, intercellular adhesion molecule-1, and vascular cell adhesion molecule-1 increased after 5 days of SAH. The NF-κB inhibitor pyrrolidine dithiocarbamate can reverse the above SAH-induced effects and reduce vasospasm after SAH, indicating that the NF-κB-mediated proinflammatory response in SAH may lead to the occurrence of cerebral vasospasm (57). The caspase inhibitor benzyloxycarbonyl-Val-Ala-Asp-fluoromethylketone was shown to reduce cerebral vasospasm on day 2 after SAH in a rabbit single hemorrhage model, which was associated with reduced IL-1b release in the cerebrospinal fluid and decreased levels of caspase-1 and IL-1b in macrophages infiltrating the subarachnoid space (58).

### 2.3 Endothelial dysfunction and cerebral vasospasm

The endothelium plays a critical role in regulating vascular tone, blood flow, and tissue perfusion. Endothelial cells produce and release a variety of bioactive molecules, including NO, endothelin-1 (ET-1), prostacyclin, and ROS, which together regulate the contraction and relaxation of smooth muscle cells in the vascular wall (59). Endothelial damage impairs the production of vasodilators such as NO and promotes vasoconstriction through the release of ET-1 and ROS (60). The pathophysiology of EC in the delayed phase of aneurysmal SAH involves a complex interaction of cerebral vasospasm, microthrombosis, and inflammation, all of which contribute to the morbidity and mortality of this disease. Iuliano et al. (61) demonstrated that endothelial dysfunction plays a key role in the development and persistence of cerebral vasospasm after SAH, as evidenced by altered vascular responses to acetylcholine and calcimycin. The process begins with EC dysfunction, which leads to smooth muscle contraction, inflammation, and altered vascular responses. Blood in the subarachnoid space, especially free hemoglobin, is a major contributor to this cascade. Hemoglobin damages neurons and ECs, leading to NO loss and ET-1 increase (62). In addition, ET-1 not only causes vasoconstriction, but also promotes inflammation and smooth muscle cell proliferation, which thickens the vessel wall, leading to vasospasm and EC damage (63). Activated immune cells clear blood products but also release inflammatory mediators that further damage the endothelium, leading to vasoconstriction, blood-brain barrier disruption, and cerebral infarction (64).

### 2.4 Smooth muscle hypercontraction and cerebral vasospasm

Vascular smooth muscle hypercontraction is the main cause of cerebral vasospasm after SAH. Numerous studies have shown that RhoA and its effector Rho-kinase (ROCK) play an important role in the regulation of Ca^2+^-independent smooth muscle contraction (65–67). The RhoA-ROCK pathway mainly regulates the phosphorylation level of myosin light chain of myosin II by inhibiting myosin phosphatase and contributes to Ca^2+^ sensitization in agonist-induced smooth muscle contraction. During SAH, a large number of red blood cells flow into the subarachnoid space. The hemolysis of these red blood cells releases hemoglobin, ET-1, cytokines, and thromboxane A2, all of which may activate G protein-coupled receptors and RhoA-ROCK signaling pathways. Activation of the ROCK signaling pathway leads to decreased MLCP activity and increased MLC phosphorylation, which in turn leads to relative enhancement of MLCK activity and contraction of vascular smooth muscle (68). When G protein-coupled receptors are activated, phospholipase C is activated. This enzyme catalyzes the hydrolysis of a molecule called phosphatidylinositol 4,5-bisphosphate in the cell membrane, producing inositol 1,4,5-triphosphate (IP_3_) and diacylglycerol (DAG). IP_3_ promotes the release of calcium from intracellular calcium stores, leading to an increase in intracellular Ca^2+^ levels. The surge in Ca^2+^ activates MLCK, which promotes the phosphorylation of myosin and induces the interaction between actin and myosin, ultimately leading to vasoconstriction [24]. In addition, DAG, a metabolic byproduct of PLC, also activates PKC. Activated PKC reduces MLCP activity, thereby enhancing MLCK activity and promoting vasoconstriction (69).

## 3 Role of sympathetic nervous system in cerebral vasospasm

Sympathetic perivascular nerve fibers originate from the superior cervical ganglion and innervate cerebral blood vessels, and their activation leads to vasoconstriction. Sympathetic nerve activation is considered to be an important trigger of cerebral vasospasm (70, 71). Recently, several studies have shown that sympathetic nerve-mediated vasoconstriction is one of the key mechanisms of vasospasm (24, 72, 73). In addition, sympathetic nerve activation is also associated with endothelial dysfunction and inflammatory response.

### 3.1 Sympathetic nerve activation and vasoconstriction

One of the most consistent findings observed in the literature is the increase in SNS activity, specifically the increase in norepinephrine levels, after SAH (24, 25, 74, 75). Both experimental animal models and human studies have shown that the release of norepinephrine into the blood and cerebrospinal fluid (CSF) is associated with the development and severity of cerebral vasospasm (76–78). Elevated norepinephrine concentrations have been detected in the CSF of SAH patients, and elevated norepinephrine induces vasoconstriction after binding to α-adrenergic receptors on vascular smooth muscle cells (79). This effect is particularly pronounced in cerebral vessels, which have a higher sensitivity to adrenaline signals (80).

The key aspect of SNS involvement in cerebral vasospasm is the phenomenon of sympathetic hyperactivity, which is commonly observed after SAH (24, 75, 81). Sympathetic hyperactivity refers to the sustained, exaggerated activation of the SNS that leads to prolonged catecholamine release and vasoconstriction. This hyperactivity arises due to several factors, including the acute stress of SAH, central nervous system injury, and dysregulated feedback mechanisms such as impaired baroreceptor function (24). Studies have shown that patients with SAH can exhibit higher-than-normal sympathetic activity, and this heightened state of SNS arousal contributes to the development of vasospasm (75).

### 3.2 Sympathetic nerve activation and endothelial dysfunction

In addition to direct effects on vascular smooth muscle, the SNS also indirectly contributes to vasospasm through the disruption of endothelial function. The endothelium is crucial for maintaining vascular tone by releasing vasodilators including NO and prostacyclin, which counterbalance the vasoconstrictive effects of sympathetic stimulation (82). After SAH, endothelial dysfunction occurs due to the inflammatory response, ROS, and other factors. As a result, the ability of the endothelium to dilate cerebral vessels is impaired, and the vasoconstrictive effects of sympathetic activation become more pronounced (12).

A study by Neuschmelting et al. investigated the role of ET-1 and NO in cerebral vasospasm after SAH. It finds elevated ET-1 levels in cerebrospinal fluid and reduced NO metabolites in basilar arterial plasma, both linked to cerebral vasospasm occurrence in a rabbit model of SAH (83). This endothelial dysfunction, coupled with sympathetic activation, leads to a heightened and sustained vasoconstrictor effect, contributing to cerebral vasospasm. Furthermore, sympathetic-mediated neuroinflammation acerbates endothelial injury, further impairing the vasodilatory capacity of cerebral vessels. In addition, sympathetic activation also leads to increased production of ET-1, a potent vasoconstrictor, through the stimulation of endothelin receptors (84). The enhanced release of ET-1 further amplifies the vasoconstrictor response in the cerebral arteries, leading to prolonged and potentially damaging vasospasm (85). Clazosentan, an endothelin receptor antagonist, has been widely investigated for the prevention of cerebral vasospasm in patients with aSAH (86, 87). Recently, a study from Japanese research group showed clazosentan's effectiveness in reducing vasospasm-related morbidity and all-cause mortality after aneurysmal SAH in Japanese patients (88).

### 3.3 Sympathetic nerve activation and inflammatory response

The acute increase in intracranial pressure and subsequent cerebral ischemia following SAH triggers widespread activation of the SNS (36, 89). This activation has profound effects on both the central nervous system (CNS) and peripheral organs, exacerbating inflammation and secondary brain injury. Understanding the interplay between SNA and inflammation in the setting of SAH could provide new insights into therapeutic strategies.

The inflammatory response is another critical component of cerebral vasospasm. After SAH, inflammatory mediators such as cytokines, prostaglandins, and ROS contribute to endothelial dysfunction and vascular smooth muscle contraction (51, 90). Sympathetic activation can amplify this inflammatory response through the release of neuropeptides, including substance P and neurokinin A (91), which enhance vascular permeability and promote further endothelial injury. This vicious cycle of inflammation and sympathetic activation may contribute to the persistence of cerebral vasospasm. Additionally, sympathetic activation primes microglia, the resident immune cells of the CNS, to adopt a pro-inflammatory phenotype (92). Systemic inflammation driven by SNA extends beyond the CNS, with evidence of heightened inflammatory markers in peripheral blood and organ dysfunction, such as acute lung injury and renal impairment, often complicating SAH management (93). This leads to increased production of ROS and further neuroinflammation. Future research is needed to focus on elucidating the precise mechanisms linking SNA to inflammation.

## 4 Sympathetic nerve system inhibition and cerebral vasospasm

SNS is a part of the autonomic nervous system and controls many involuntary body functions, including vascular tone and heart rate. Studies have shown that excessive sympathetic activity promotes vasoconstriction and reduces cerebral blood flow (94, 95), which can lead to vasospasm in SAH patients. Considering the role of the SNS in cerebral vasospasm, researchers have investigated strategies to modulate SNS activity as potential therapeutic approaches. Sympathetic nerve blockade has been shown to reduce the severity of vasospasm following SAH (96, 97). In the following sections we will discuss the role of sympathetic nerve block in preventing cerebral vasospasm and its application prospects.

### 4.1 Sympathetic nerve block is a therapeutic strategy

Based on the role of sympathetic activation in cerebral vasospasm, several therapeutic strategies targeting the SNS have been explored. Several studies have shown that sympathetic nerve block helps reduce the severity of vasospasm (97, 98). The stellate ganglion is a collection of sympathetic nerve cells located at the junction of the C7 and T1 vertebrae in the neck (99). It is part of the sympathetic trunk and is responsible for providing sympathetic innervation to the upper extremities, head, neck, and thoracic organs (100). The stellate ganglion controls the tone of blood vessels, including those in the cerebral and peripheral circulations, by releasing norepinephrine upon sympathetic activation (101). The stellate ganglion block (SGB) can inhibit this sympathetic activity and induce vasodilation. SGB is a percutaneous procedure involving the injection of a local anesthetic into the stellate ganglion and has been used to treat many conditions, such as chronic pain, anxiety, ventilation, and diabetes (102–104). It has also recently been proposed as a potential treatment for patients at risk for vasospasm after SAH (97). By blocking the stellate ganglion, SGB reduces sympathetic nerve flow to the cerebral arteries, decreases sympathetic nerve activity, and thus leads to vasodilation and improves cerebral blood flow, low mortality and complication rates, suggesting its importance as a therapeutic intervention for vasospasm after SAH (97). Although the number of studies evaluating SGB as a preventive measure is limited, the encouraging results highlight the importance of future research.

### 4.2 SGB modulates the cerebral vasoconstriction and vasodilation

In the context of vasospasm, sympathetic overactivity exacerbates the condition by causing persistent vascular smooth muscle contraction. By targeting the stellate ganglion, SGB aims to decrease sympathetic tone and prevent further vasoconstriction (105). In cerebral vasospasm, particularly following SAH, SGB can potentially reduce the severity of vasospasm, alleviate ischemia, and improve neurological outcomes. A study showed that the use of endothelin-β receptor agonists or hexamethonium at the stellate ganglion can increase the secretion of nitric oxide synthase at its nerve endings. A series of studies have shown that parasympathetic nerve excitation can weaken the inhibitory effect of nitric oxide synthase; NO production will decrease under conditions of high sympathetic nerve activity; the plasma content of calcitonin gene-related peptide is significantly increased after SGB; there are also reports that norepinephrine can inhibit the release of calcitonin gene-related peptide and α-adrenergic receptor blockers can stimulate the release of calcitonin gene-related peptide. Animal experiments on rats undergoing extracorporeal circulation showed that SGB can significantly reduce the concentration of ET-1 in serum and hippocampal tissue, while the concentration of calcitonin gene-related peptide (CGRP) is significantly increased. A meta-analysis showed that CGRP significantly increases the diameter of animal brain blood vessels (106).

### 4.3 SGB reduces inflammatory response

**Figure 2:**
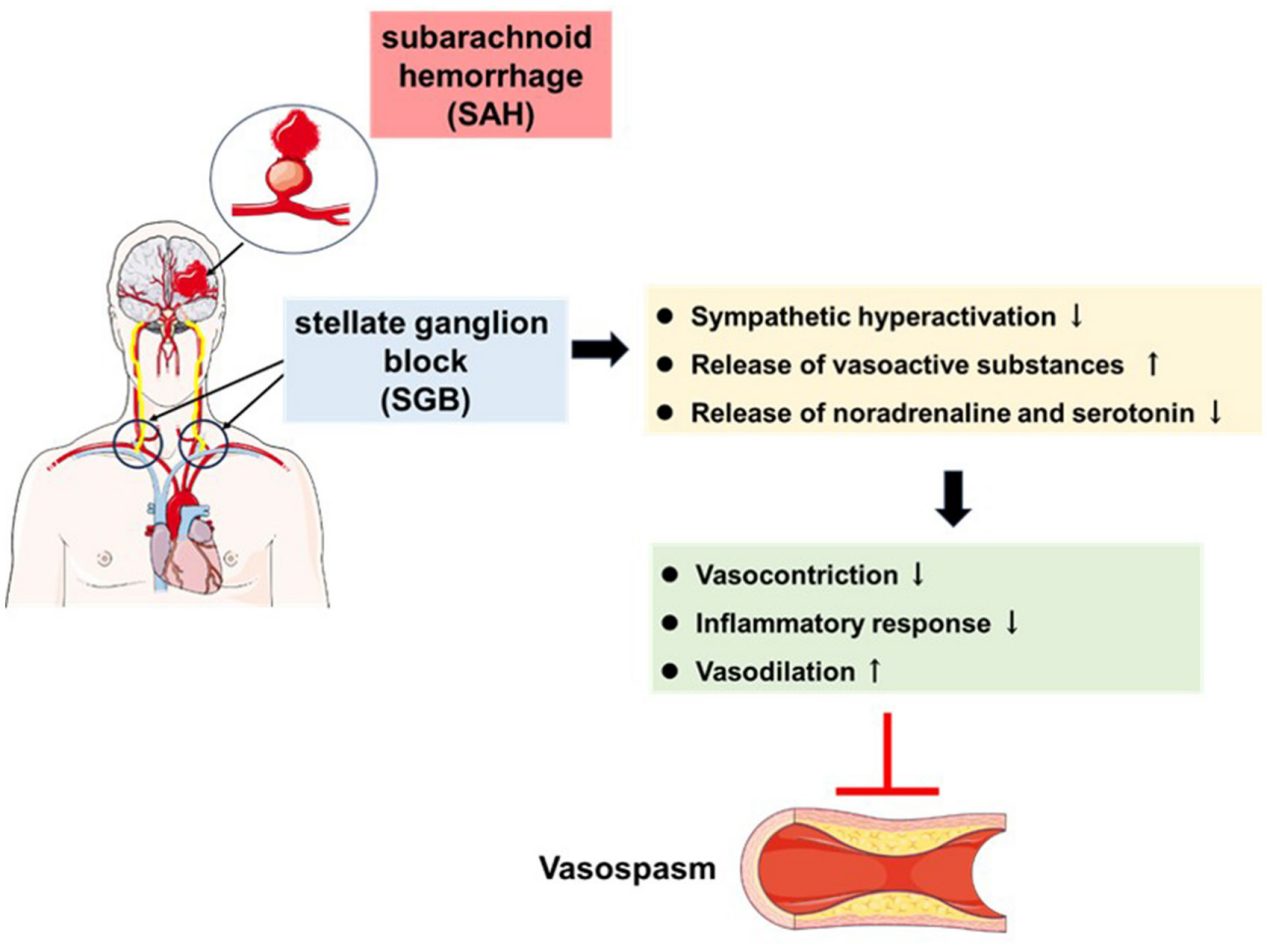
Illustration of stellate ganglion block (SGB) for targeting cerebral vasospasm after subarachnoid hemorrhage (SAH).

In 2021, a study from Zhang et al. (107) randomly divided 102 patients into NSGB and SGB groups, and monitored serum inflammatory cytokines (IL-1β and IL-6) and brain injury markers (neuron-specific enolase and S100 calcium-binding protein β). The results showed that the levels of brain injury markers in the SGB group were significantly lower than those in the NSGB group, and the manifestations of neurological deficits were also significantly less, such as hemiplegia and cognitive impairment (107). Another study on patients with traumatic brain injury found that SGB could significantly reduce the level of NF-κB p65 in the patient's serum (108), which suggests that SGB has an inhibitory effect on the TLR4/NF-κB pathway. At the same time, SGB also reduced the concentration of IL-6 in the serum, further alleviating the inflammatory response (108). After SGB treatment, sympathetic nerve activity can be reduced, the release of vasoactive substances can be increased while the release of vasoconstrictive substances can be reduced, cerebral vasoconstriction can be reduced, inflammatory response can be reduced, and cerebral blood flow velocity can be significantly reduced (Figure 2). According to existing studies, some positive results have been achieved, with low mortality and complication rates, highlighting its importance as a therapeutic intervention for vasospasm after SAH. Although the number of studies evaluating SGB as a preventive measure is limited, the encouraging results emphasize the importance of future research.

## 5 Clinical applications and efficacy of SGB

### 5.1 Clinical efficacy and safety of SGB in cerebral vasospasm after SAH

Several studies have investigated the potential of SGB in reducing the incidence and severity of cerebral vasospasm following SAH. Some studies have reported significant improvements in cerebral blood flow, reduced vasospasm severity, and enhanced patient outcomes (27, 98, 109). A systematic review and meta-analysis of 8 studies on SGB in subarachnoid SAH patients revealed favorable outcomes in 52% of patients, with low complication (2%) and mortality rates (13%). SGB reduced cerebral blood flow velocity, showing promise as a treatment for vasospasm, though more research is needed (97). However, another study from Samagh et al. evaluated the efficacy and safety of SGB in relieving cerebral vasospasm in aneurysmal SAH patients. After SGB, significant reductions in middle cerebral artery peak systolic velocity, mean flow velocity, and Lindegaard ratio were observed. Neurological improvement occurred in 25% of patients, but effects on microvasculature were limited (28). Future research, particularly larger and more rigorous trials, is required to definitively establish its clinical efficacy.

SGB is generally regarded as a safe procedure when performed by skilled clinicians. Local anesthetics are commonly used to block nerve transmission and relieve pain (110). The dose of local anesthetic administered during SGB depends on the clinical condition being treated, the patient's response, and the specific anesthetic used. Here, we summarize the use of local anesthetics in the treatment of SGB after SAH based on published reports (Table 1). The choice and dose of anesthetic depends on the patient's physical condition, the desired duration of action, and the technique used. However, as with any invasive intervention, potential risks exist, including Horner's syndrome (due to sympathetic blockade in the cervical sympathetic chain), local infection, hematoma, and unintentional nerve damage (111, 112). These complications are rare and typically manageable. Ensuring appropriate patient selection, employing expert techniques, and maintaining diligent monitoring are essential to minimize risks.

**Table 1:** The volume of local anesthetics for stellate ganglion block (SGB) treatment following SAH.

**Condition**	**Anesthetic agent**	**Volume of local anesthetic**	**Reference**
Early brain injury (EBI) after SAH	Ropivacaine (0.375%)	8 ml	(107)
Delayed cerebral ischemia (DCI) after SAH	Ropivacaine (0.5%)	20ml	(120)
Aneurysmal SAH	Bupivacaine (0.5%)	10 ml	(30)
Aneurysmal SAH	Bupivacaine (0.5%)	10 ml	(28)
Aneurysmal SAH	Ropivacaine (0.5%)	8 mL	(98)
Ischemic encephalopathy or cerebral vascular disease	Levobupivacaine (0.375%)	8 ml	(109)
Refractory cerebral vasospasm after aSAH	Ropivacaine (0.5%)	5 ml	(114)

### 5.2 Recurrence rate of after SGB treatment

Although SGB can provide immediate relief of vasospasm, recurrence of vasospasm after blockade remains a concern, especially when the underlying pathophysiology of vasospasm has not been fully resolved. The recurrence of vasospasm may be related to delayed or incomplete blockade or when the blockade fails to significantly reduce the improvement of sympathetic tone in the affected vascular territory. A study by Saket Sanghai MBBS et al. compared single-injection vs. continuous-infusion SGB for treating ventricular arrhythmia (VA) storm. Their results showed that continuous-infusion SGB resulted in a significantly greater reduction in VA burden compared to single-injection SGB, with similar safety profiles and fewer repeat procedures required (113). These findings suggest that repeated blocks may help to more effectively control the condition by persistently reducing sympathetic activity. The success of SGB depends on the skill of the practitioner and the accuracy of the block. Improper execution of the block may result in incomplete or transient effects, which may increase the likelihood of recurrence. In addition, SGB combined with other therapies, such as vasodilators or antihypertensive medications, may reduce the recurrence rate of vasospasm (114). Further research and large-scale trials are needed to better understand the long-term efficacy of SGB and improve treatment options to minimize recurrence and improve outcomes for SAH patients.

### 5.3 SGB as an adjunct therapy to endovascular treatments in cerebral vasospasm

Endovascular treatments, such as intra-arterial infusion of vasodilators (e.g., nimodipine, papaverine), are commonly employed to manage severe vasospasm following SAH (115, 116). However, the recurrence of vasospasm and its associated complications can still pose significant challenges. Intra-arterial infusion of vasodilators and balloon angioplasty are widely used as the primary treatment for severe cerebral vasospasm (19, 117, 118). However, these interventions have several limitations. Surgery may carry risks such as arterial dissection, bleeding, or vascular injury (119). In addition, the effectiveness of endovascular treatments may be limited in cases of diffuse vasospasm or small vessels that are difficult to access. SGB is a neuroablative technique that targets the sympathetic nervous system, has been explored as an adjunctive therapy to endovascular interventions. Andrea Bortolato et al. reported a case report of using continuous SGB during early DCI. They describe a patient with early DCI after SAH, where standard nimodipine endovascular treatment failed to restore normal cerebral perfusion (120). Recently, a randomized controlled trial by Jian Zhang et al. investigated the effect of SGB on patients with SAH (107). Their results showed that SGB significantly reduced EBI markers and cerebral vasospasm, leading to better neurological outcomes and prognosis compared to standard care (107).

### 5.4 SGB for cerebral vasospasm: overview of indications

SGB is a sympathetic blockade therapy commonly used to treat pain syndromes, but has also been used to treat cerebral vasospasm, particularly aneurysmal SAH (28, 30, 107). Although the utility of SGB is unclear compared with balloon angioplasty or intra-arterial vasodilators, SGB has attracted attention due to its non-invasive nature, low risk, and potential to improve cerebral blood flow (114). SGB is considered the best option before invasive interventions such as balloon angioplasty, especially when digital subtraction angiography is not available or delayed. The case report suggests that SGB may benefit patients with poor perfusion in the posterior circulation (98). Additionally, when invasive medical treatments fail or are temporarily unsuitable, SGB can be considered as a rescue method for DCI (120, 121).

## 6 Conclusions

Cerebral vasospasm following SAH is a major contributor to morbidity and mortality. To date, no definitive prevention or effective treatment strategies have been established. The interaction between sympathetic nerve activity and cerebral vasospasm is complex, with sympathetic activation leading to both vascular smooth muscle contraction and endothelial dysfunction. A better understanding of the mechanisms by which sympathetic nerve activation influences cerebral vasospasm could provide new opportunities for therapeutic intervention. SGB is a sympathetic nerve block procedure that has shown potential therapeutic benefits in the management of cerebral vasospasm. SGB has been demonstrated to reduce cerebral vascular tone, promote dilation of cerebral blood vessels, and attenuate inflammatory responses, thereby improving cerebral circulation and alleviating vasospasm. These effects may ultimately contribute to better clinical outcomes in patients with SAH-related vasospasm. Despite its promise, research on the use of SGB for the prevention and treatment of cerebral vasospasm following SAH remains limited. Further basic and clinical studies are required to explore its efficacy, optimal application, and long-term outcomes. Only with such research can the full therapeutic potential of SGB in this context be realized.
